# Human Nail Plate Modifications Induced by Onychomycosis: Implications for Topical Therapy

**DOI:** 10.1007/s11095-014-1562-5

**Published:** 2014-11-22

**Authors:** A. Baraldi, S. A. Jones, S. Guesné, M. J. Traynor, W. J. McAuley, M. B. Brown, S. Murdan

**Affiliations:** 1 Department of Pharmaceutics, UCL School of Pharmacy, University College London, 29 -39 Brunswick Square, London, WC1N 1AX UK; 2 Institute of Pharmaceutical Science, King’s College London, Franklin-Wilkins Building, 150 Stamford Street, London, SE1 9NH UK; 3 Department of Pharmacy, School of Life and Medical Sciences, University of Hertfordshire, College Lane, Hatfield, Hertfordshire AL10 9AB UK; 4 MedPharm Ltd, Unit 3 Chancellor Court, 50 Occam Road, Surrey Research Park, Guildford, GU2 7YN UK

**Keywords:** barrier, fungal, nail, onchomycosis, topical drug delivery

## Abstract

**Purpose:**

Through the characterisation of the human onchomycotic nail plate this study aimed to inform the design of new topical ungual formulations.

**Methods:**

The mechanical properties of the human nail were characterised using a Lloyd tensile strength tester. The nail’s density was determined via pycnometry and the nail’s ultrastructure by electron microscopy. Raman spectroscopy analysed the keratin disulphide bonds within the nail and its permeability properties were assessed by quantifying water and rhodamine uptake.

**Results:**

Chronic *in vivo* nail plate infection increased human nailplate thickness (healthy 0.49 ± 0.15 mm; diseased 1.20 ± 0.67 mm), but reduced its tensile strength (healthy 63.7 ± 13.4 MPa; diseased 41.7 ± 5.0 MPa) and density (healthy 1.34 ± 0.01 g/cm^3^; diseased 1.29 ± 0.00 g/cm^3^). Onchomycosis caused cell-cell separation, without disrupting the nail disulfide bonds or desmosomes. The diseased and healthy nails showed equivalent water uptake profiles, but the rhodamine penetration was 4-fold higher in the diseased nails using a PBS vehicle and 3 -fold higher in an ethanol/PBS vehicle.

**Conclusions:**

Onchomycotic nails presented a thicker but more porous barrier, and its eroded intracellular matrix rendered the tissue more permeable to topically applied chemicals when an aqueous vehicle was used.

**Electronic supplementary material:**

The online version of this article (doi:10.1007/s11095-014-1562-5) contains supplementary material, which is available to authorized users.

## Introduction

Onychomycosis refers to the infection of the nail unit by fungi. It constitutes 40% of all reported nail disorders (1). Responsible fungi include dermatophytes (most frequently *Trichophyton rubrum, Trichophyton interdigitale and Trichophyton mentagrophytes*), moulds (*Scytalidium* spp., *Scopulariopsis* spp., *Fusarium* spp., *Acremonium* spp., *Onychocola canadensis*) and yeasts (*Candida* spp.) (2). Onychomycosis may be treated by both oral and topical routes; with topical treatment reducing the risks of hepatotoxic side effects associated with some systemic antifungals. However, topical medication appears to provide relatively low cure rates (typically up to 30%) and relatively long treatment times (12 months or longer) (3). One reason for the low cure rates of topical therapy is thought to be the inability of active agents to penetrate the nail plate, but the studies that propose this generally use healthy tissue to study chemical penetration and there remains a need to conduct such studies in the presence of the disease (4–6).

The onychomycotic nail is visually very different to the healthy nail. Clinically it presents as a thickened, crumbling barrier in advanced disease. These properties have been previously characterized qualitatively using electron microscopy (7–11) and are thought to be caused by enzymatic tissue degradation by keratinolytic proteinases that are released by the invading organisms during an onychomycotic episode (12–17). The keratinolysis which occurs during the infection is also thought to be accompanied by sulfitolysis, which results, according to the data generated in hair samples, in the breakage of disulfide bonds (18). However, to date, the thickness, the density, the sulfur bonding and barrier properties of diseased nails have not been quantified. Therefore, a strong link between onychomycotic induced nail plate changes and nail plate barrier properties has not yet been established.

The aim of this investigation was to understand how onychomycosis-induced structural changes influenced nail plate barrier function. To achieve this aim structural (surface morphology, cell layer integrity, cell-cell linkages, nail thickness, density), mechanical (tensile strength, elasticity, fracture strain) and chemical properties (disulfide bonds) of healthy and diseased human nails were studied. This data was interpreted alongside a comparison of rhodamine B uptake (19–22) and nail hydration characterisation in healthy and infected nails in order to try and determine how the invading organisms altered the passage of chemicals into the tissue.

## Materials and Methods

### Chemicals

Formalin solution (10%, neutral buffered), tris (2-carboxyethyl) phosphine hydrochloride (≥98%), rhodamine B (95%), 2-propanol (≥99.5%), sulfuric acid (95–98%), were reagent grade and purchased from Sigma Aldrich (Dorset, UK). Absolute ethanol (AnalaR, Normapur), was supplied by VWR. Hydrochloric acid was supplied by Fisher Scientific (Loughborough, UK). High-purity nitrogen gas was supplied by BOC (UK). Calibrated ChubTur® diffusion cells were kindly provided by MedPharm Ltd.

### Nail Collection and Storage

Healthy nail clippings were supplied by volunteers (ethics approval, REC/B/10/01 School of Pharmacy, University of London, UK) and diseased nail samples were supplied by podiatrists and dermatologists (ethics approval, PHAEC/09–24 University of Hertfordshire; 12/YH/0381, NRES Committee Yorkshire & The Humber, Sheffield, UK). Dirt and debris were removed from the healthy nails, they were washed with water (23–26) and stored at room temperature. Diseased nails were sealed in vials and stored at 4°C as the fungi viability is maintained, but growth prevented under these conditions (27). Artificial nail infection was achieved as previously elaborated (26) using *T. rubrum* with an incubation period of 21 days. All nail comparisons were matched throughout the study *i.e.*, finger nails were compared with finger nails and toe nails were compared to toe nails, therefore the source of the nail was noted in the text only when it was relevant to the data interpretation.

### Electron Microscopy

The specimens were fixed overnight in 10% formalin solution (pH 7 in phosphate buffer), then dehydrated in a series of ethanol solutions at ascending volume ratios (from 50 to 100%) and cut into three pieces of approximately 2 mm width. The samples were mounted on SEM stubs (AGAR Scientific, Cambridge, UK), sputter coated with 20 nm of gold (Au) (Quorum Q150 S/C) and viewed with a FEI QUANTA 200 F scanning electron microscope (Eindhoven, Netherlands). A total of 30 samples were prepared and grey scale images at ×500 and ×5000 magnification were captured. The SPIP 6.0.12 software package (Image Metrology A/S, Denmark) was used to estimate the total pore surface coverage ratio, the average surface coverage per pore and pore size distribution at each magnification (×500/×5000) and condition (healthy/diseased). Transmission electron microscopy sections were cut using a Reichart-Jung Ultracut-E ultramicrotome (Leica Microsystems Ltd, Knowlhill, UK). They were mounted on copper grids, contrasted with uranyl acetate and lead citrate and examined on a FEI Tecnai 12 transmission microscope operated at 120 kV (FEI UK Ltd, Cambridge, UK). Images were acquired with an AMT 16,000 M digital camera (Woburn, MA, United State of America). TEM images were then processed using ImageJ 1.46 software (National Institutes of Health, Bethesda, Maryland, United State of America). The width of desmosomes and intercellular spaces were calculated by measuring the area of the desmosome or intercellular space and dividing it by its perimeter on the image analysis software.

### Physical Characterisation

Thickness measurements were made at the edges and at the centre of the human nail plate using an electronic micrometer at room temperature at ambient relative humidity (40–60%) (28,29). Nail density was recorded using nitrogen gas on a Multi-Pycnometer (Quantachrome Corporation, Florida, United State of America). The mechanical properties of the human nail were assessed using a uniaxial pull to break test with a LF500 Lloyd material testing machine fitted with metal fibre clamps (Fareham, UK). For each Stress/Strain profile the strength parameters: yield stress (σ_y_), Young’s modulus (E), ultimate tensile strength (UTS), fracture stress (σ_f_) and the strain parameters: yielding strain (e_y_), ultimate tensile strain (e_uts_) and fracture strain (e_f_) were calculated.

### Raman Analysis

A Raman microscope (XploRA™, HORIBA Scientific, UK) with a 785 nm (infra-red) laser excitation and a spectral resolution of 4 cm^−1^ was employed to characterize the S-S and –SH groups of healthy and diseased nail clippings from two positions for each nail sample. All the spectra were subjected to baseline correction and normalisation using the Amide I band, 1628–1679 cm^−1^ as the reference peak. The healthy and diseased nail plate spectra were compared to a control with cleaved disulfide bonds, which was generated by exposing healthy nails to a solution of 0.8 M of (tris (2-carboxyethyl) phosphine (TCEP) in water (pH 1.23) for 3 days (to cleave the nail disulfide bonds).

### Nail Penetration Assays

The rhodamine uptake was based on the method of Potsch *et al.* (19). The nail clippings were weighed and incubated in a 0.2 mM rhodamine B aqueous solution (pH 3.74) for 30 min at 32°C. Incubation of the samples in a 4:1 (*v/v*) isopropanol:0.1 M sulfuric acid solution at 60°C for 1 h was subsequently used to extract the rhodamine B that had been taken up into the nail tissue. The rhodamine content of the 4:1 *v/v* isopropanol:0.1 M sulfuric acid solutions was determined using a Lambda 25 UV/Visible spectrophotometer (Perkin Elmer, UK) at 556 nm. Nail water uptake was determined through exposure to distilled water and gravimetric analysis. The rhodamine transport studies were performed using the ChubTur® cells (MedPharm Ltd, Guildford, UK) with 3 mm × 3 mm nail clippings (dorsal nail surface area of 0.05 cm^2^). The receiver fluid (0.5 ml) was either phosphate buffered saline (PBS) or 1:1 PBS/ethanol (*v/v*). Penetrant solutions were prepared and used at saturated concentrations (*i.e.* a fine suspension) to maintain a constant thermodynamic activity regardless of the nature of the donor/receiver fluids. Sink conditions were maintained during the studies. The donor chambers were sealed with Parafilm® for the duration of the experiment to prevent evaporation of penetrant solutions. The receiver fluid was sampled at predetermined time points. At each time point, before sampling, the cells were visually inspected to check for leaks (observed via solvent back diffusion) and inverted 3 times to thoroughly mix the liquids. A 500 μL aliquot of receiver solution was then transferred into disposable UV/visible microcuvette. The sample was replaced with fresh receiver fluid. As no rhodamine B permeation through the nail samples was detected after 5 weeks the cells were emptied; the nails samples were dismounted, briefly rinsed with ethanol and patted dry with a clean tissue. Rhodamine B was extracted from the nail samples with a 4 mL solution of 4:1 (*v/v*) isopropanol:0.1 M sulfuric acid at 60°C repeatedly until the extraction solution contained no detectable rhodamine. The rhodamine content of the washing solutions was determined using UV/VIS spectrophotometry as described in the rhodamine B uptake studies.

### Statistical Analysis

Results are presented as the mean ± standard deviation and Student’s *t*-test were performed to test for significant differences between samples unless stated otherwise in the text. When ANOVA is cited as the statistical test one way ANOVA was used (IBM SPSS for Windows, version 11.0). A level of 0.05 was taken as significant for all the tests employed in this work.

## Results and Discussion

### Nail Thickness and Density

The thickness of healthy nails collected in this study was found to be between 0.3–1 mm which agrees with previous reports (30). The diseased nails donated by patients were significantly thicker than healthy nails (*p* ≤0.05, ANOVA, Table I). A value for diseased nail thickness could not be found in the literature, but the observed increase in thickness corresponded with the visual observations reported in previous clinical studies (31). The increased nail thickness was found to be consistent across the whole nail clipping (*p* >0.05), but it was not observed in early infected nail plates *in vitro*. The diseased nail density was lower compared to that of healthy nails, which suggested they had a more porous structure (*p* ≤0.05, Mann–Whitney test, Table I). The healthy nail density was in accordance with previously published results, which suggested the measurement methods were appropriate (32), but again no density data was found in the literature for the diseased nail plate. Image analysis of scanning electron micrograph data showed the diseased nails contained larger pores than healthy nails (comparisons made at the same magnification, Table I, Figure S4), which supported the density measurements.

**Table I:** Comparison of Nail Physical Properties

Characteristic	Healthy nail	Diseased nail	*In vitro* infected
Pore size (μm^2^)	0.78 ± 0.29	1.52 ± 0.59	1.92 ± 1.24
Nail thickness (mm)	0.49 ± 0.15	1.20 ± 0.67	0.31 ± 0.08
Nail density (g/cm^3^)	1.34 ± 0.01	1.29 ± 0.00	1.26 ± 0.00

data represents mean ± one standard deviation (*n* = 3)

### Nail Structure

**Fig. 1:**
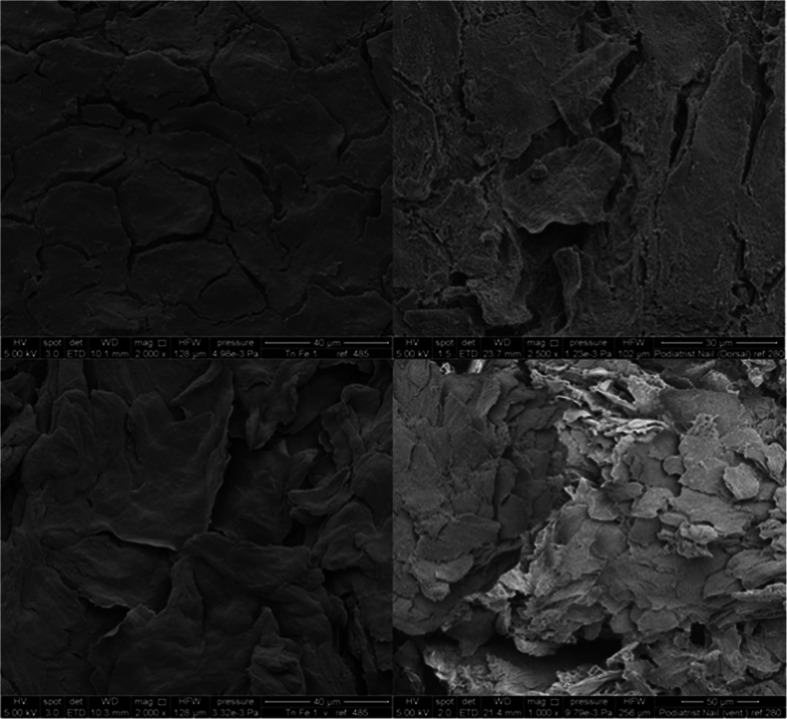
SEM micrographs of a healthy (*left*) and diseased nail (*right*), dorsal layer (*top*); ventral layer (*bottom*).

Scanning electron micrographs of healthy nails displayed a relatively smooth dorsal and ventral surface. The cells that could be observed appeared tightly packed and the nail plate presented visually as a highly confluent barrier (Fig. 1). In contrast, the diseased nails contained fragmented regions, they showed cell lifting and cell-cell separation (Fig. 1, cell separation confirmed in cross sections Figure S1, supplementary information). It has previously been reported that dermatophytes, which have proteolytic (13,14) and lipolytic (15) activities, can invade the nail plate and perturb cell-cell interactions (33). Therefore, the separation of cells in the diseased nails was attributed to the alteration of the intercellular nail matrix as a result of the protease action. Similar changes to the nail structure were observed in electron microscopy images of onychomycotic nails donated from patients and those artificially infected *in vitro,* albeit the latter showed visibly more damage than the former (Figure S3). Some differences in the *in vitro* and *in vivo* infected nails were anticipated as infecting the nail *in vitro* with only *T.rubrum* for 21 days does not exactly mimic the conditions the nails are exposed to *in vivo*. In this study the purpose of using data from the *in vitro* -infected nails was to bench mark the changes that occurred *in vivo* with an infection that had been controlled in the laboratory setting such that the action of the infection upon the nail properties could be better understood. Transmission electron micrograph images showed the desmosome links - with a structure similar to those previously reported (31) were present in the infected nail structure and that they were intact; their size did not change (11.53 ± 2.86 nm for healthy and 8.29 ± 1.72 nm for diseased nails *P* > 0.05)), this supported the suggestion that the disease process modified the extracellular matrix without removing the cell-cell attachments (Figure S2).

### Nail Tensile Strength

**Fig. 2:**
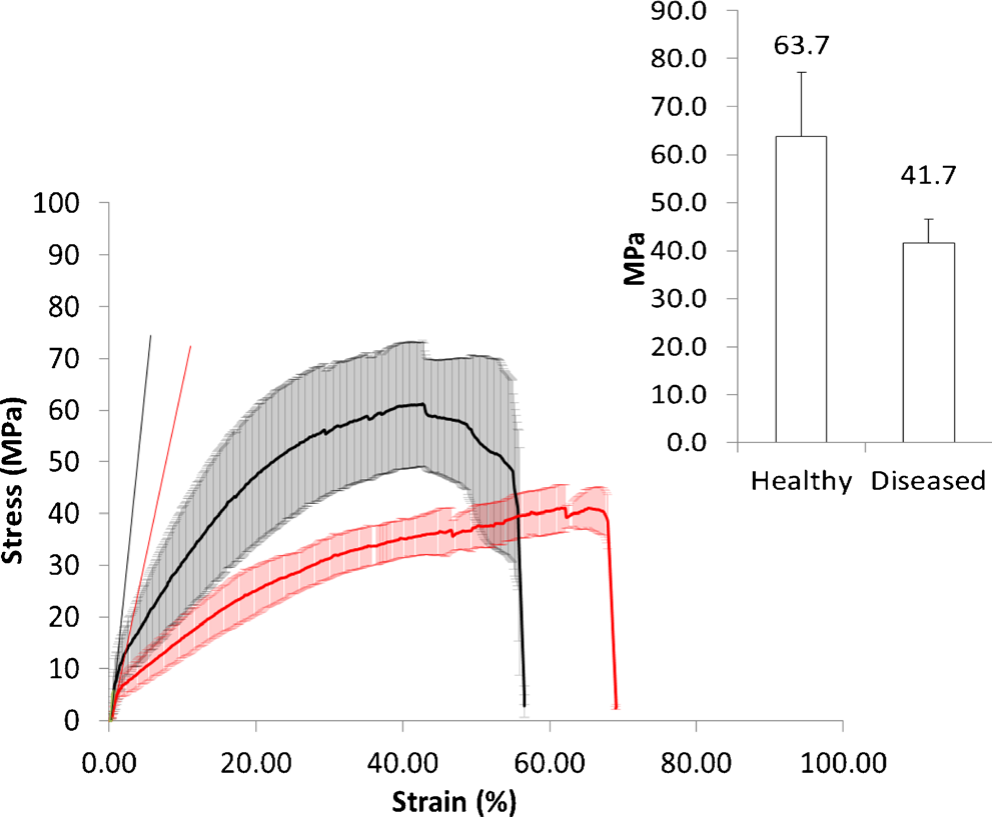
Stress/strain profiles of healthy (*black trace*) vs *in vivo* infected diseased nails (*red trace*), inset: comparison of ultimate tensile strength of the nails.

The stress/strain profiles of keratin fibers typically show three distinct regions when subjected to mechanical strength analysis. These regions are defined as: pre-yield (linear elastic), pseudo-yield and post yield (stiffening) regions, representing the stretching of α keratin fibers, the transition of keratin from the α to the β form and the stretching of β keratin fibers respectively (34–36). These three regions were not observed in this study (Fig. 2), which suggested the nail matrix had a pronounced effect on the tensile properties of the tissue (37). The diseased nail plates exhibited a significantly lower initial stiffness (*E* =0.8 ± 0.3 vs *E* =1.6 ± 0.5 GPa, *p* ≤0.05), yield stress (σ_y_ =5.1 ± 1.8 vs 9.3 ± 2.1 MPa, *p* ≤0.05) and ultimate tensile strength (UTS =41.7 ± 5.0 vs 63.7 ± 13.4 MPa, *p* ≤0.01, data calculated from Fig. 2) compared to healthy nails. The changes were interpreted as being a consequence of the nail infection altering the matrix in which nail cells were embedded rather than the sulfur links of the keratin fibers as all the strain related indices e.g. yielding strain (e_y_ =1.0 ± 0.3 healthy and 1.0 ± 0.2 diseased, *p* >0.05), fracture strain (σ_f_ =56.5 ± 18.1 healthy and 69.1 ± 3.7 diseased, *p* >0.05), ultimate tensile strain (e_uts_ =48.3 ± 15.9 healthy and 67.9 ± 3.2 diseased, p >0.05) and fracture stress (e_f_ =43.6 ± 20.0 healthy and 38.4 ± 6.3 diseased p >0.05) were unchanged. Subsequent disulfide bond analysis supported this conclusion.

### Nail Disulfide Bonding

**Fig. 3:**
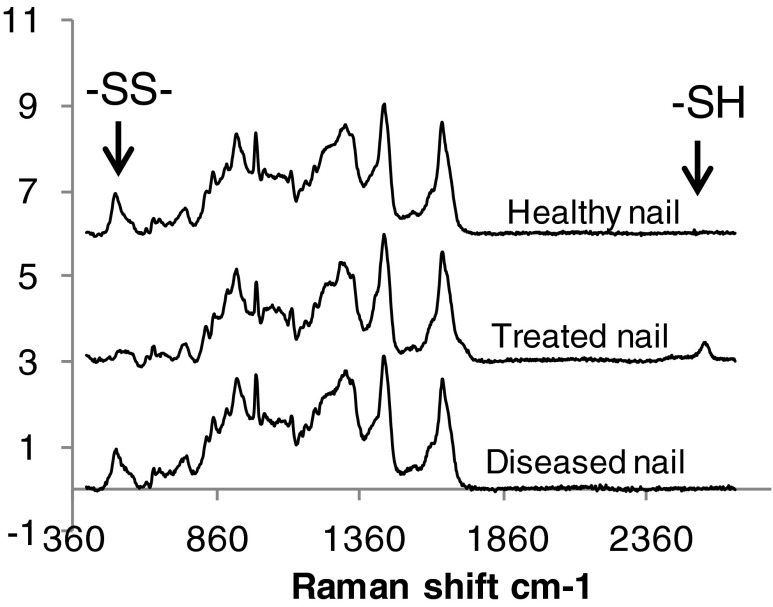
Raman spectra of nail dorsal surfaces when presented as healthy nails, TCEP-treated nails and diseased nails.

An intact keratin disulfide bond (S-S group) vibration displays a Raman spectra peak at 430–550 cm^−1^ whilst a cleaved disulfide (S-H group) displays a peak at 2550–2600 cm^−1^. The spectra of the healthy nails analysed in this work showed the S-S peak, in accordance with previously published work (38–40), but no -SH peak (Fig. 3). Treatment of the healthy nails with TCEP, a phosphine-based molecule with the ability to break disulfide bonds (41)), reduced the S-S peak height and generated a -SH peak (at 2575 cm^−1^, Fig. 3). This confirmed the ability of the Raman method to detect the breakdown of the S-S bond in the nail. The diseased nail also showed an S-S peak, but not an –SH peak and this suggested that the keratin disulfide links that were present within the nail were not significantly perturbed by the organism invasion associated with onchomycosis. It was interesting to note that position of S-S peaks in the dorsal layer (ca. 510 cm^−1^) suggested a more stable S-S bond (42,39) compared to the ventral surface (Table S1). In previous work it was suggested that the infection could start from the under surface of the nail (nail bed and nail plate’s ventral surface) (43) and the Raman data suggests this could be a weak point compared to the dorsal surface of diseased nails.

### Nail Hydration and Barrier Properties

**Fig. 4:**
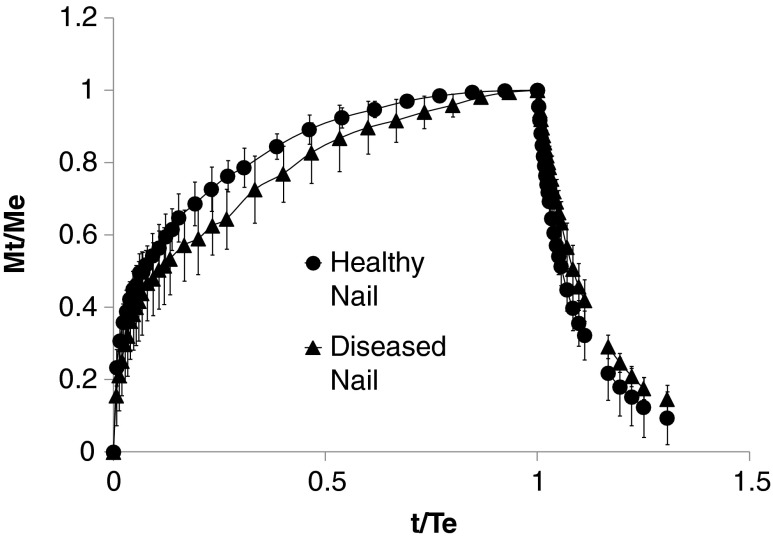
Water content (Mt) normalised to water content at equilibrium (Me) against time (t) normalised to the time at equilibrium (Te) of heathy versus diseased nails (Mean ± sd, *n* = 3; 2 cycles per n).

The water uptake and loss by diseased and healthy nails was similar. The maximum % water uptake was between 16 and 24% for healthy and 17 and 25% for diseased tissue (Fig. 4). The water uptake equilibrium time (time to reach saturation) ranged between 90 to 150 min and the water loss time (time to reach the nail initial drying weight) ranged between 110 to 350 min with no statistical difference between healthy and diseased water uptake and loss profiles (*p* >0.05, repeated measures ANOVA). Previous work on the absorption/desorption capacity of water in healthy human nails has shown that chemical treatment results in structural nail damage and modifies the response of the tissue to water (44). Therefore, the data reported in the current work suggests that the thickening of the nail plate, in response to onychomycosis infection, counteracts the nail matrix changes, *i.e.*, the increased porosity and reduced density, to present a barrier that responds to water hydration in a similar manner to healthy nail tissue.

**Fig. 5:**
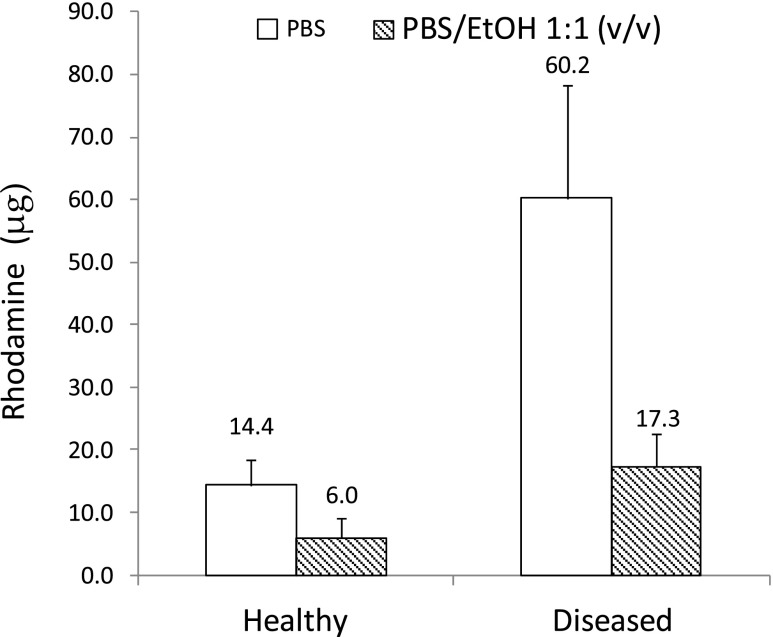
Transport of Rhodamine through the human nail plate using two different application vehicles, an aqueous phosphate buffer (PBS) at pH 7.4 and an aqueous phosphate buffer at pH 7.4 mixed with ethanol (EtOH).

The Raman data, which indicated the nail plate maintained its overall integrity, even after the nail infection had digested some of the intracellular material to make cell-cell separation visible on the electron micrographs, was supported by acute single application rhodamine uptake studies (see Figure S5 and (22)). However, it was thought wise to expand these studies using a second experimental design, which determined the effect of the nail barrier upon prolonged exposure to the rhodamine. When rhodamine solutions were applied to the dorsal surface of the nail plate repeatedly over a period of 5 weeks, mimicking topical drug application in clinical practice, the molecule did not pass through the tissue, but a difference was observed in the manner in which the diseased and healthy nails took up the dye (Fig. 5). Transport into the nail was found to be ca. 3-fold greater in diseased nails compared to healthy nails when a PBS:ethanol mixture was employed as the administration vehicle (*p* ≤0.05) and this difference increased to 4-fold when rhodamine was administered using PBS alone (*p* ≤0.05). The relatively high molecular weight of rhodamine compared to the antifungal agents typically applied to the nail was probably an important contributing factor to its slow movement through the tissue (45,46), but the differential transport into the diseased and healthy nails was thought to be representative of a change in the barrier properties of the nails when subjected to the chronic re-application of the aqueous rhodamine solution. As the maximum hydration of the nail plate took place at ca. 2 h it was possible that the differential uptake of rhodamine between the healthy and diseased plate was because the nails remained in a hyper hydrated state for such a long time frame when nails were exposed to the dye for extended periods of time. Although using a single chemical probe limits the scope of this conclusion, rhodamine was considered to be a molecule that should be particularly sensitive to changes in the tissue barrier properties and a good model for antifungal agents. The reason for this is that it is ionised at physiological pH’s (Marvin Sketch, Version 5.5, ChemAxon Ltd.), like the two topical antifungals amorolfine and ciclopirox, thus it is susceptible to both electrostatic and hydrophobic interactions with the nail tissue (47). The superior ability of an aqueous vehicle alone to influence penetration reflects the previously reported greater permeation of molecules from aqueous, rather than non-aqueous or water/co-solvent mixtures (48). This could have important implications for the development of new topical formulations because it suggests that aqueous films should be used to generate topical formulations rather than the alcohol based systems that are currently in clinical use (3).

## Conclusion

This investigation suggests that the *in vivo* thickening of nail plate that occurred as a result of onychomycosis infection maintains the water homeostasis of the underlying tissue despite the invading organism’s ability to digest the intercellular matrix and render the tissue less dense with larger pores. The keratin in the nail appeared to maintain its S-S bonds and its overall strength despite the presence of the disease. The nail plate mechanical properties indicated its rigidity had changed and this was thought to be a consequence of the micro-organism’s disruption of the matrix in which the nail cells were embedded, an effect that was also observed visually in the electron micrographs as cell-cell separation. A preliminary study using rhodamine suggested that the barrier integrity of diseased nails appeared to be greater only when the nails were forced to hydrate beyond their natural state through contact with a polar solvent over several days. This finding could provide a basis for an improved therapeutic intervention either through the development of a new topical medicine or the adaptation of current treatment regimens to encourage hyper-hydration of the nail plate during onychomycotic infection. However, further studies are required to better understand which types of molecules could benefit from the enhanced uptake into the diseased nail through its forced hydration with water.

## Electronic supplementary material

Below is the link to the electronic supplementary material.

ESM 1 (DOC 10762 kb)
