# Supplementary material for: Human Nail Plate Modifications Induced by Onychomycosis: Implications for Topical Therapy
Source: Pharm Res. 2014 Nov 22;32(5):1626–33. doi: 10.1007/s11095-014-1562-5 (PMC4381097; doi:10.1007/s11095-014-1562-5)
Supplement: Supplementary file 1 — (DOC 10762 kb) [file 11095_2014_1562_MOESM1_ESM.doc]

# Human nail plate modifications induced by onychomycosis: implications for topical therapy

# Supplementary Material

~~
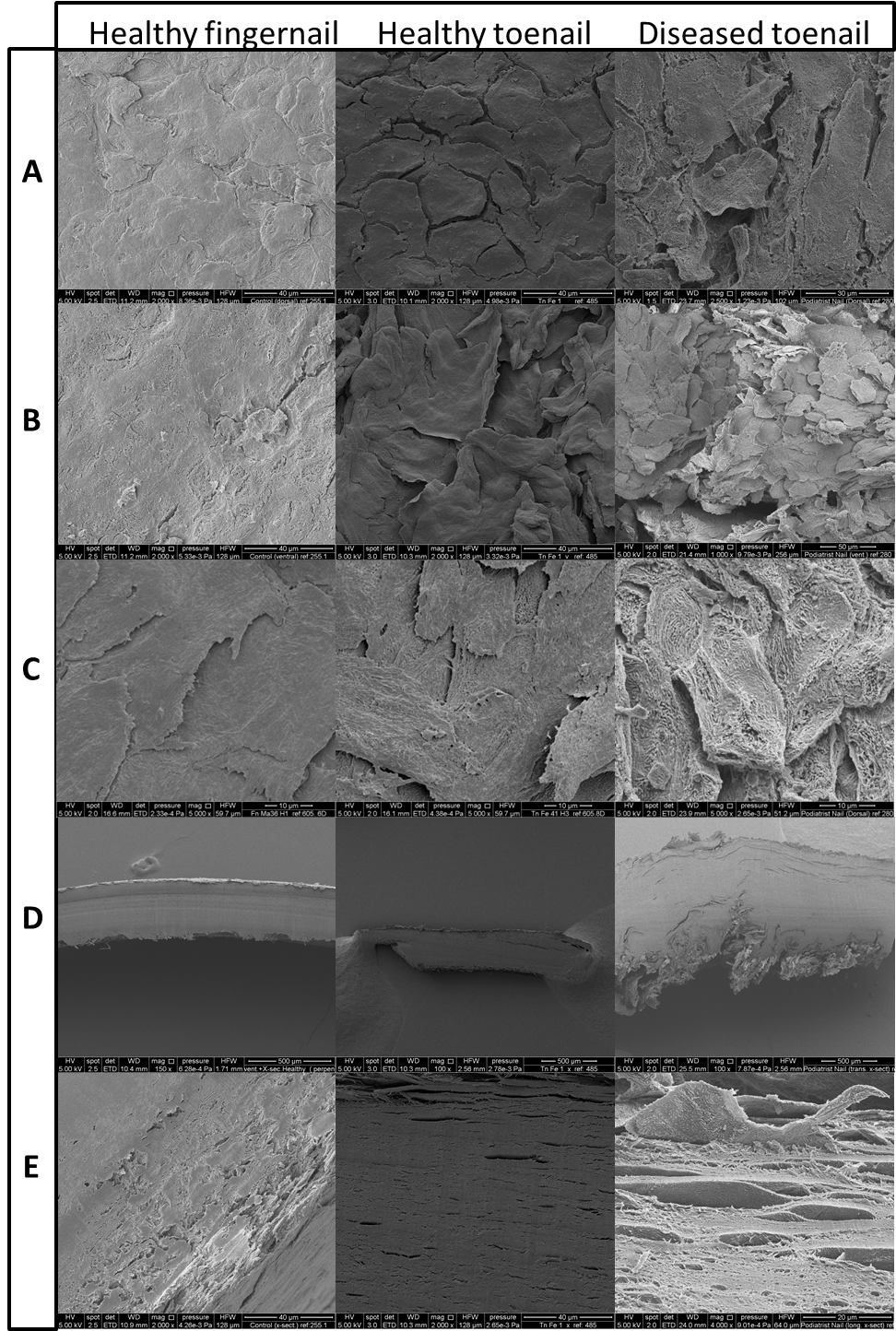
~~

Figure S1: SEM cross-sectional images of a healthy nail (left column) and a diseased nail (right column).


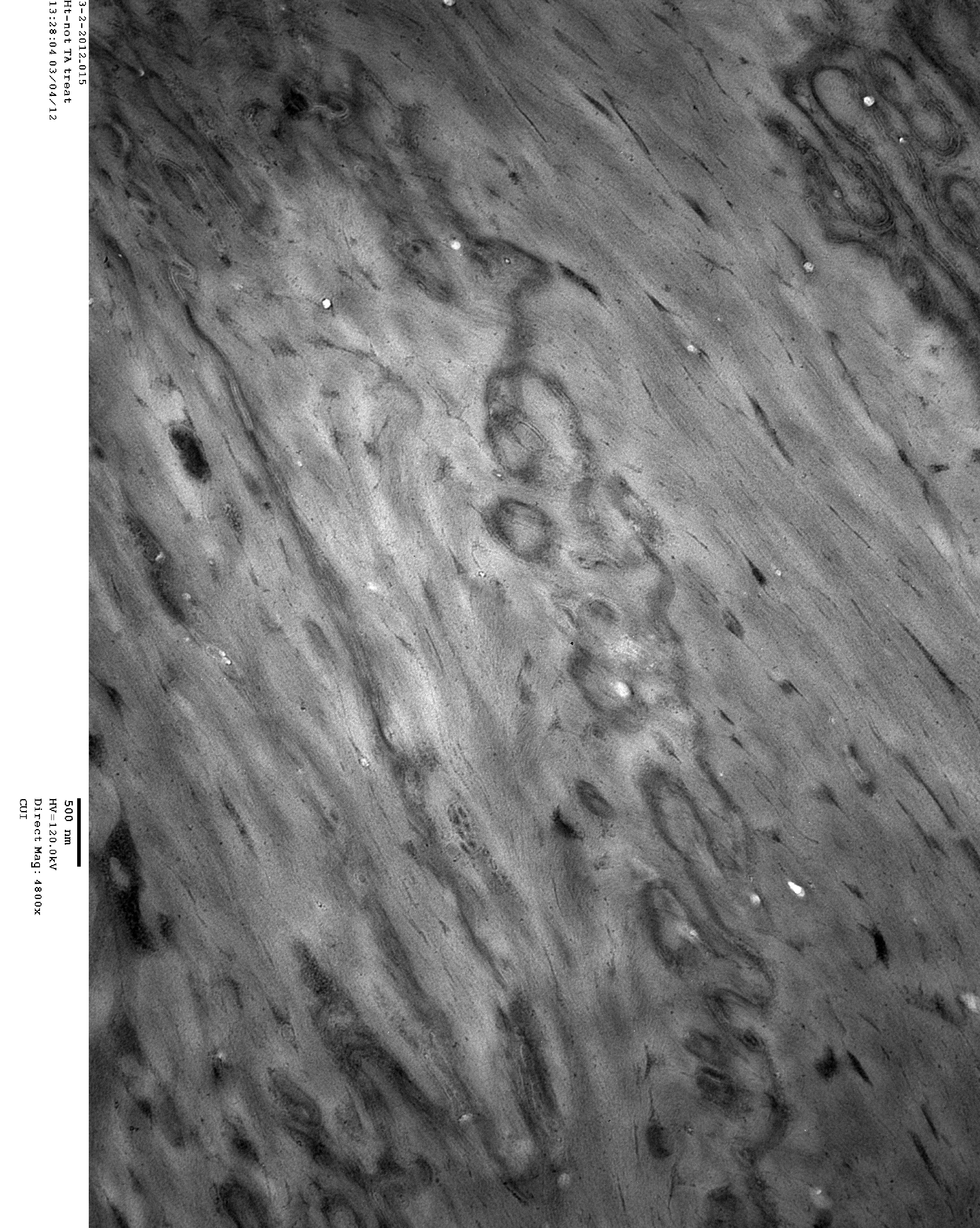


Figure S2: TEM image of a healthy nail. A indicates an anchoring knot. B indicates a desmosome; X4800

**A**

**B**


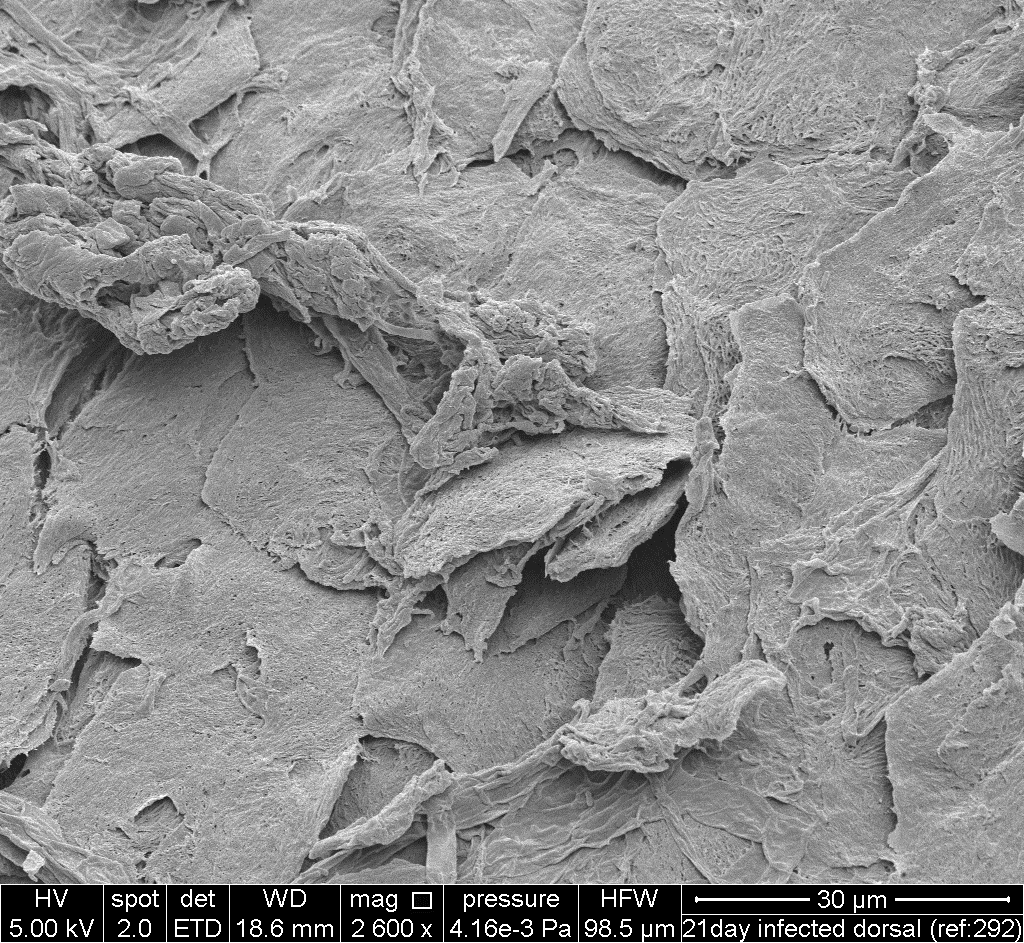

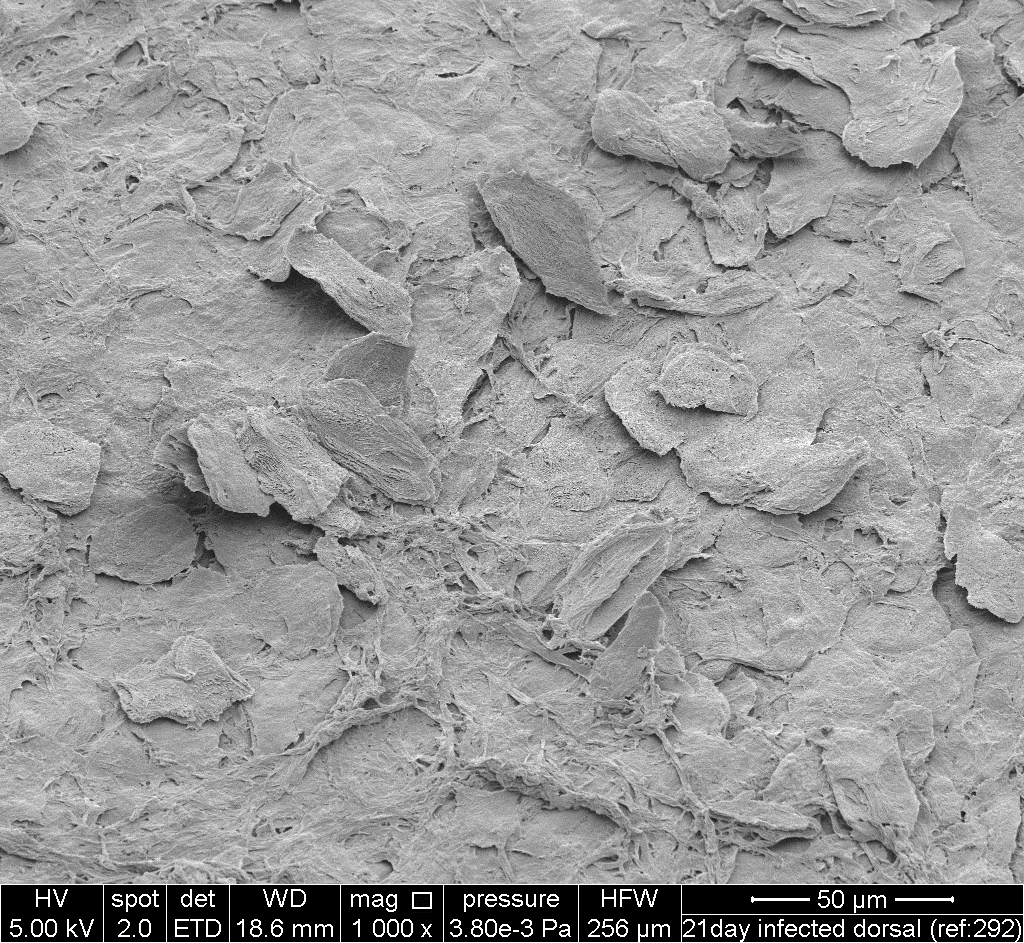


Figure S3: SEM dorsal surface images of a diseased nail that was infected in vitro.


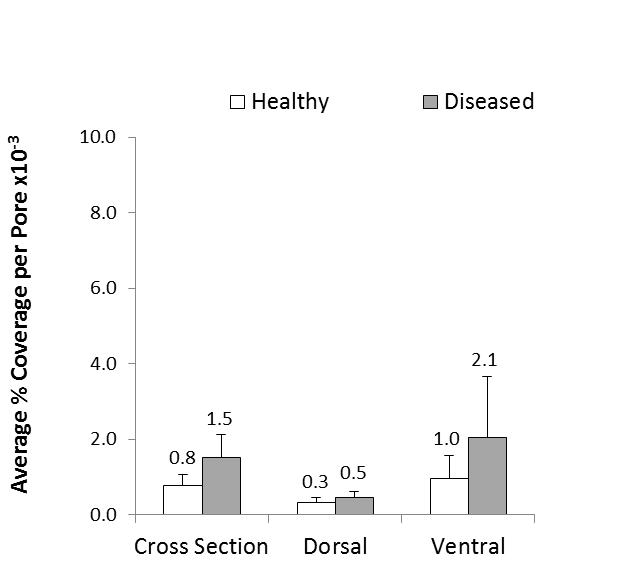


Figure S4: Average Percentage of Surface coverage per Pore of the dorsal, ventral and cross-section surfaces of healthy and diseased nails at x 500 magnification using the SEM images.

Figure S5: Uptake of Rhodamine into the human nail plate from an aqueous solution upon 30 minutes of exposure. Means of n=3 ± 1 standard deviation.

Table S1: Raman shifts and intensity of S-S band in healthy and diseased nails. Means and ± one standard deviation are shown (n=4 for healthy nails; n=5 for diseased nails).

| **Type of nail** | **Nail surface** | **S-S Raman Shift** | **S-S intensity** |
| --- | --- | --- | --- |
| Healthy nails | Dorsal | 508.09 ± 0.57 | 0.98 ± 0.13 |
|  | Ventral | 515.26 ± 6.14 | 0.62 ± 0.35 |
| Diseased nails | Dorsal | 509.51 ± 1.58 | 0.82 ± 0.16 |
|  | Ventral | 522.35 ± 19.14 | 0.72 ± 0.25 |
|  |  |  |  |
